# Molecular Differences in Hepatic Metabolism between AA Broiler and Big Bone Chickens: A Proteomic Study

**DOI:** 10.1371/journal.pone.0164702

**Published:** 2016-10-19

**Authors:** Aijuan Zheng, Wenhuan Chang, Guohua Liu, Ying Yue, Jianke Li, Shu Zhang, Huiyi Cai, Aijun Yang, Zhimin Chen

**Affiliations:** 1 Feed Research Institute/Key Laboratory of Feed Biotechnology of Ministry of Agriculture, Chinese Academy of Agricultural Sciences, Beijing 100081, China; 2 Key Laboratory of Pollinating Insect Biology of Ministry of Agriculture, Institute of Apicultural Research, Chinese Academy of Agricultural Sciences, Beijing 100081, China; 3 CSIRO Agriculture, Brisbane 4067, Australia; Virginia Commonwealth University, UNITED STATES

## Abstract

Identifying the metabolic differences in the livers of modern broilers and local chicken breeds is important for understanding their biological characteristics, and many proteomic changes in their livers are not well characterized. We therefore analyzed the hepatic protein profiles of a commercial breed, Arbor Acres (AA) broilers, and a local dual purpose breed, Big Bone chickens, using two-dimensional electrophoresis combined with liquid chromatography-chip/electrospray ionization-quadruple time-of-flight/mass spectrometry (LC-MS/MS). A total of 145 proteins were identified as having differential abundance in the two breeds at three growth stages. Among them, 49, 63 and 54 belonged to 2, 4, and 6 weeks of age, respectively. The higher abundance proteins in AA broilers were related to the energy production pathways suggesting enhanced energy metabolism and lipid biosynthesis. In contrast, the higher abundance proteins in Big Bone chickens showed enhanced lipid degradation, resulting in a reduction in the abdominal fat percentage. Along with the decrease in fat deposition, flavor substance synthesis in the meat of the Big Bone chickens may be improved by enhanced abundance of proteins involved in glycine metabolism. In addition, the identified proteins in nucleotide metabolism, antioxidants, cell structure, protein folding and transporters may be critically important for immune defense, gene transcription and other biological processes in the two breeds. These results indicate that selection pressure may have shaped the two lines differently resulting in different hepatic metabolic capacities and extensive metabolic differences in the liver. The results from this study may help provide the theoretical basis for chicken breeding.

## Introduction

Over the past few decades, China’s broiler industry has transformed from traditional farming to the intensive farming model. At the same time, the rapid development of the broiler industry is associated with poor meat quality and negative feedback. Commercial broilers are characterized by fast growth and a high feed-to-weight conversion rate; however, excessive abdominal fat deposition and poor meat quality are becoming the major concerns in the poultry industry [1]. Fortunately, indigenous chicken breeds produce better quality meat and the cross between indigenous chickens and broilers may provide a way to overcome these problems [2].

Broiler chickens demonstrate differences in fat deposition, indicating the importance of genetic factors in fat deposition [3]. Unlike mammals, de novo fatty acid synthesis (lipogenesis) in birds occurs mainly in the liver [4]. Previous studies have revealed that fat and lean lines of broilers differed in the metabolism of very low-density lipoprotein [5, 6] and fatty acid in the liver [7, 8]. Moreover, the liver is a vital organ in chickens which carries out multiple metabolic roles. Profiling of liver proteins abundance can aid in the understanding of variations in poultry liver metabolism.

The Arbor Acres (AA) broiler is a well-known commercial breed in the poultry industry. It is featured by a large size, rapid-growth rate, high feed-conversion rate and strong adaptability, but possesses less favorable meat quality and excessive abdominal fat [9]. Gene expression analysis demonstrated that the AA breed had dysregulated lipid metabolism and other cellular pathways compared to a Chinese breed with high meat quality [10]. In contrast, Big Bone Chickens, which originated in the Liaoning province of China, is a meat-and-egg dual-purpose local breed and famous for its big size and large eggs as well as delicious and nutritious meat [11]. Thus, these two breeds can offer suitable models to study the difference in the relationship between liver metabolism and meat quality. This study aims to identify the molecular mechanism of metabolic differences between modern broilers and local chicken breeds, in an attempt to provide the theoretical basis for improving meat quality in commercial chickens.

## Materials and Methods

AA broilers and Zhuanghe Big Bone chickens were raised, sampled and slaughtered in the Nankou experimental farm of the Feed Research Institute, Chinese Academy of Agricultural Sciences (CAAS), Beijing, China. The care and use of all birds in this experiment was approved by the Animal Care and Use Committee of the Feed Research Institute of CAAS. The proteomics analysis was conducted in the Feed Research Institute, CAAS.

### Reagents

Tris-base, ammonium persulfate, sodium dodecyl sulfate (SDS), N,N,N’,N’-tetramethylethylenediamine (EDTA), sodium bicarbonate (NH_4_HCO_3_), glycine, agarose, urea trichloroacetic acid (TCA), and formic acid were purchased from Sigma (St. Louis, USA). Bio-lyte was purchased from Bio-Rad (Hercules, CA, USA). Acrylamide, N, N’-methylenebisacrylamide, bromophenol blue, coomassie brilliant blue G-250, thiourea, 3-[(3-cholamidopropyl) dimethylammonio]-1-propanesulfonate (CHAPS), glycerol, and bovine serum albumin were purchased from Amresco (Solon, OH). Dithiothreitol (DTT) and iodoacetamide were purchased from Merck (Darmstadt, Germany). Trypsin was purchased from Roche (Basel, Switzerland), and trifluoroacetic acid and acetonitrile were from J. T. Baker (Phillipsburg, NJ). Lastly, dipotassium phosphate (K_2_HPO_4_), monopotassium phosphate (KH_2_PO_4_), sodium chloride (NaCl), sodium hydroxide (NaOH), and acetone were purchased from Beijing Shiji Co. (Beijing, China).

### Care and management of chickens

Seventy-two each of AA broilers and Zhuanghe Big Bone chickens (1-day-old) were purchased from the Xingwang Chicken Company. (Liaoning, China). Each group had 6 replicates and each replicate had 12 individuals. The chickens were reared from 0 to 42 days and fed with corn-soybean starter (days 0–21) and grower (days 22–42) diets. All chickens were subject to 23 h light and 1 h dark on days 0–7, and 20 h light and 4 h dark thereafter in accordance with the AA broiler and Zhuanghe Big Bone Chicken Management Guides. The room temperature was maintained at 33–35°C on days 0–3, at 32–34°C on days 4–7 and gradually reduced to the maintenance temperature of 20°C by day 42. The relative humidity was kept at 70% during the first week and thereafter at about 60%.

### Liver protein extraction

At the age of 2 weeks, 4 weeks, and 6 weeks, twelve chickens from each group (two birds each replicate) were randomly selected, electrically stunned, and manually slaughtered within 5 min [12] Livers were collected and washed in cold saline solution (0.9% NaCl) to clear blood and other contaminants, and were frozen immediately in liquid nitrogen and stored at -80°C for further processing.

Protein preparation was performed as described previously with some modifications [13]. To avoid erroneous conclusions due to individual variations, the same quantity of proteins from the liver of four chickens were pooled as a biological replicate, and three biological replicates were acquired for each group. Briefly, the frozen liver tissues were manually ground into fine powders using a mortar and pestle in liquid nitrogen, and were then homogenized in PBS (pH 7.6) containing 32.5 mM K_2_HPO_4_, 2.6 mM KH_2_PO_4,_ and 400 mM NaCl. The mixture was sonicated for 2 min and centrifuged at 14,000 *g* for 10 min at 4°C. The supernatant was stored for later use. The pellets were washed in PBS (pH 7.6) and were homogenized in lysis buffer (LB, 9 M urea, 2 M thiourea, 4% CHAPS, 20 mM Tris-base, 30 mM DTT, and 2% Bio-lyte, pH 3–10). The homogenate was then sonicated for 2 min and centrifuged at 14,000 *g*, 4°C for 10 min. The supernatant was transferred to a tube containing PBS protein extract. TCA was added to a final concentration of 10% and then the mixture was kept on ice for 10 min for protein precipitation and desalting. Subsequently, the mixture was centrifuged twice at 14,000 *g* for 10 min at 4°C and the pellets were suspended in loading buffer, sonicated for 1 min and adjusted to pH 7.0. About four-times volume of acetone was added into the protein mixture, the mixture stored at -20°C for 2 hours for protein precipitation and desalting. Next, the mixture was centrifuged and the protein pellets were resolved in LB. The protein extract was stored at -70°C for further use. Protein concentration was determined according to the Bradford method [14] against a bovine serum albumin standard curve, at 595 nm in a spectrophotometer DU800 (Beckman Coulter, Los Angeles, CA).

### Two dimensional gel electrophoresis

Three gels were independently carried out with each biological replicate sample. Each 500 μg protein sample suspended in lysis buffer was mixed with rehydration buffer (8 M urea, 2% CHAPS, 0.001% bromophenol blue, 45 mM DTT, 0.2% Bio-lyte pH 3–10). The mixture was loaded onto a 17 cm IPG strip (pH 3–10, linear, Bio-Rad). Isoelectric focusing (IEF) was performed at 18°C according to manufacturer’s instructions (Protean IEF Cell, Bio-Rad). Before the second dimension of electrophoresis, the IPG strips were first equilibrated in equilibration buffer I [6 M urea, 0.375 M Tris-HCl (pH 8.8), 20% glycerol, 2% SDS, 2% DTT] for 15 minutes and then in equilibration buffer II [6 M urea, 0.375M Tris-HCl (pH 8.8), 20% glycerol, 2% SDS, 2.5% iodoacetoamide] for another 15 minutes. After equilibration, the strip was transferred to a SDS polyacrylamide gel, 12% T separating gel (1.00 mm). The second dimension of electrophoresis was performed in a Protean II Xi Cell (Bio-Rad) at 25 mA/gel for about 5 h.

### Image acquisition and statistical analysis

Gels were fixed in 50% (v/v) ethanol and 10% (v/v) acetic acid solution overnight and then stained with coomassie brilliant blue G-250. Three technical replicate gels were scanned to acquire images and evaluate reproducibility. Images were imported into PD Quest V 8.0 (Bio-Rad) for further analysis. The abundance of each spot was expressed as %Vol (spot volume / total volumes of all spots resolved in the gel). The average values from three independent experiments were calculated and considered to be statistically significant with p<0.05 and at least a 2-fold change.

### Mass spectrometry analysis

The protein spots were excised and digested with 10 ng/μl trypsin (Roche) for MS analysis as previously reported [15]. The peptides were analyzed by liquid chromatography-chip/electrospray ionization-quadruple time-of-flight/mass spectrometry (QTOF G6520, Agilent Technologies). Tandem mass spectra were retrieved using MassHunter software (Version B. 02. 01, Agilent Technologies). Before MS/MS data searching, a peak-list was generated by Mascot Distiller software (Version 3. 2. 1. 0, Matrix Science). MS/MS data were searched with Mascot 2.2 (Matrix Science) against NCBInr (release date on March, 2015). Carbamidomethylation (C) and oxidation (M) parameters were selected as fixed and variable modifications, respectively. The other parameters were: taxonomy, all entries; enzyme, trypsin; missed cleavages, 1; peptide tolerance, ± 20 ppm; MS/MS tolerance, ±0.02 Da. A total of 6,649,798 sequences and 2,279,950,795 residues in the database were searched. When the identified peptides matched to multiple members of a protein family, the match was determined based on a higher Mascot score, and differential patterns of protein spots on 2-DE gels. Protein identifications were accepted if they had a probability score greater than 95% and contained at least two identified peptides with maximum peptide coverage (S1 Table).

### Bioinformatics analysis of differentially expressed proteins in the liver between AA broiler and Big Bone chickens

The ClueGo plug-in of Cytoscape software (http://cytoscape.org/) with the Gene Ontology (GO) database (released June 2015) and the Kyoto encyclopedia of genes and genomes (KEGG) database (released October 2015) were used for functional and pathway enrichment analysis of the identified differential abundance proteins. The significantly enriched GO terms were performed by the right-side hypergeometric statistical test, which compares the background set of GO annotations in the whole genome of Gallus gallus database. Its probability value was corrected by the Bonferroni method [16]. The protein-protein biological interaction network (BIN) of differential proteins was constructed using the Search Tool for the Retrieval of Interacting Genes (STRING) 10.0 (http://string-db.org/) [17]. The network nodes represent proteins, and the edges represent the predicted functional associations.

### Validation of proteins with differential abundance by qPCR (Real-time Quantitative PCR Detecting System)

To further understand the relationship between proteins and their encoding genes, qPCR was run for differential abundance proteins at the mRNA level. Specific primers for target genes of the identified proteins were designed using the primer BLAST of NCBI and nucleotide information in GenBank (S2 Table). Total RNA was prepared from the liver of control and treated groups using SGTriEx (SinoGene Scientific Co., Beijing, China). RNA quality and concentration were detected using Biophotometer (Eppendorf, Hamburg, Germany) and agarose gel electrophoresis (S1 Fig). cDNA synthesis with 5 μg of RNA was performed using Thermo First cDNA Synthesis Kit (SinoGene). qPCR was conducted using the StepOnePLUS(Applied Biosystems, Massachusetts, USA). The PCR was performed in a 20-μL reaction system containing 1 μL of cDNA, 0.4 μL of each primer (10 μM), 10 μL of SG Green qPCR Mix (SinoGene) and 8.2 μL of water. The fold-change was calculated with the 2 ^−ΔΔCt^ method [18]. All operation for qPCR was followed by the MIQE guide-lines [19].

## Results

### Abdominal fat and liver weight comparison between AA broiler and Big Bone chickens

Average abdominal fat percentage was significantly higher in AA broilers than that in Big Bone chickens on day 28 (p < 0.05, Table 1). Abdominal fat percentage was not significantly different between female and male Big Bone chickens, but female AA broilers had higher abdominal fat than male AA broilers of the same age (day 28). Female AA broilers had significantly higher abdominal fat than male AA broilers and Big Bone chickens of the same body weight (3.0±0.13kg). The same weight Big Bone chickens and male AA broilers showed no significant difference in abdominal fat percentage.

**Table 1:** Carcass index contrast between AA broiler and Big Bone chickens.

	Big Bone chickens		AA broiler chickens	
	male	female	male	female
Abdominal fat percentage, %				
Same age (day 28)	5.53±1.03^a^	5.20±0.91^a^	18.25±2.93^b^	24.82±2.84^c^
Same body weight (3.0±0.13kg)	6.97±2.57^a^	7.12±1.21^a^	8.08±2.82^a^	14.03±1.81^b^
Liver percentage, %				
Same age (day 28)	37.49±3.53^a^	34.45±1.22^a^	26.17±0.74^b^	26.23±1.36^b^
Same body weight (3.0±0.13kg)	23.77±2.19^a^	24.37±1.12^a^	28.59±1.56^a^	27.66±1.76^a^

**Notes:** There were significant difference between different letters of groups in the same row (P< = 0.05), but no significant difference with the same letter (p>0.05).

There was no significant difference in the liver weight between female and male chickens of these two breeds on the same day or the same body weight. Big Bone chickens and AA broilers of the same weight showed no significant difference in liver weight percentage. However, the liver weight was significantly higher in Big Bone chickens than that in AA broilers on the same day (day 28).

### Identification of proteins with differential abundance

**Fig 1:**
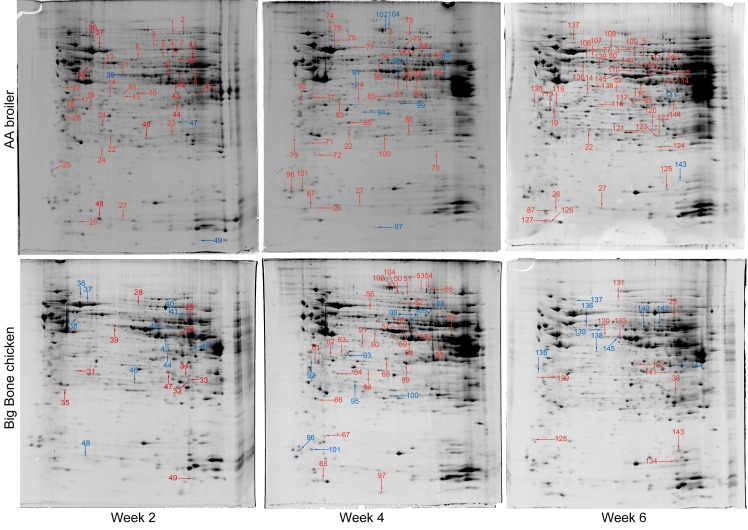
2-DE hepatic protein profiles of AA broilers and Big Bone chickens. Protein spots showing significant differences (2-fold, p < 0.05) were manually excised and identified by LC-Chip-ESI-QTOF-MS. Proteins of differential abundance with known identities were numbered and marked red or blue for increased or decreased abundance, respectively.

We compared the liver proteome between AA broiler and Big Bone chickens at three age stages using 2-DE and LC-MS/MS analysis. There were 264 to 263, 242 to 235 and 275 to 200 protein spots detected on 2-DE gels at 2 weeks, 4 weeks, and 6 weeks of age, in AA broiler to Big Bone chickens, respectively. Among them, 78, 84, and 87 protein spots showed significantly differential abundance (> 2 fold, p < 0.05) at 2, 4, and 6 weeks respectively. Subsequently, 49, 63, and 54 protein spots were identified after MS analysis at 2, 4, and 6 weeks respectively (Fig 1, Table 2). The remaining unidentified differential protein spots on 2-DE images could be due to their low abundance to produce enough spectra or their less than 95% search scores in the databases.

**Table 2:** Protein spots of differential abundance identified in the liver of AA broiler and Big Bone chickens.^a^

Spot no.	Protein name	Accession no.(NCBInr)	Symbol ID	Theoretical *M*r (kDa)/*p*I	Sequence coverage (%)	Matched/searched	Mascot score	Log _2_^ratio^ (treatment/control)		
								2 weeks	4 weeks	6 weeks
**Carbohydrate metabolism and energy production**										
4	NADP-dependent malic enzyme	gi|45383538	ME1	62.53/6.45	63	32/62	785	8.93		
5	NADP-dependent malic enzyme	gi|45383538	ME1	62.53/6.45	50	16/37	331	8.65	8.85	8.74
7	malic enzyme	gi|45383538	ME1	62.53/6.45	33	10/28	142	9.86		
10	sulfotransferase	gi|45384226	CHST3	36.33/5.89	68	22/56	739	8.50		8.17
11	ubiquinol—cytochrome c reductase	gi|50754375	UQCRFS1	53.41/6.58	6	2/5	58	9.38		
14	alpha-enolase	gi|46048768	ENO1	47.62/6.17	26	8/25	320	8.38	6.82	8.25
16	sulfotransferase	gi|45384226	CHST3	36.33/5.89	30	3/10	115	7.65		
17	sulfotransferase	gi|118088279	CHST3	36.79/5.50	8	1/4	62	9.79	9.01	
18	phosphoglycolate phosphatase	gi|71894743	PGP	33.55/5.53	19	4/5	121	8.57		
22	sepiapterin reductase	gi|50767570	SPR	29.28/5.82	45	6/13	635	8.42	8.00	8.82
23	phosphoglycerate mutase 1	gi|71895985	PGAM1	29.05/7.03	72	11/30	396	9.42		
29	electron transfer flavoprotein-ubiquinone oxidoreductase, mitochondrial	gi|71895853	ETFDH	53.61/8.99	42	7/35	969	-8.19		-9.63
44	malate dehydrogenase, cytoplasmic	gi|57530355	MDH1	36.75/6.92	54	6/32	724	1.10		
45	fructose 1,6-bisphosphatase	gi|50762393	FBP2	37.05/8.07	65	16/52	1132	1.02		
47	voltage-dependent anion-selective channel protein 1	gi|76443696	VDAC1	30.74/6.85	32	2/15	295	-1.15		
80	sorting and assembly machinery component 50 homolog	gi|118083116	SAMM50	52.47/5.99	30	2/17	540		7.37	8.84
83	galactose mutarotase (aldose 1-epimerase)	gi|118087781	GALM	38.18/6.19	18	1/6	166		7.53	
84	alcohol dehydrogenase [NADP+]	gi|57529654	ADH5	37.34/7.66	35	11/17	168		7.90	
85	L-lactate dehydrogenase B chain	gi|45383766	LDHB	36.69/7.07	40	10/13	249		8.73	9.06
87	ubiquinol-cytochrome c reductase hinge protein	gi|118094600	UQCRH	9.45/5.17	43	1/6	125		8.30	8.15
88	phosphoglucomutase-1	gi|84619526	PGM1	67.06/8.98	65	43/61	1142		1.16	
89	fumarate hydratase, mitochondrial	gi|57530433	FH	54.49/9.20	49	27/31	1098		-1.08	
98	NADP-dependent malic enzyme	gi/45383538	ME1	62.53/6.45	43	27/68	657		1.73	
102	pyruvate carboxylase	gi|45383466	PCX	128.03/6.26	61	81/135	2199		-1.32	
104	pyruvate carboxylase	gi|45383466	PCX	128.03/6.26	53	49/99	1099		-1.09	
120	carbonic anhydrase II	gi|833606	CA2	28.82/6.51	41	8/12	218			7.31
121	phosphomannomutase 2	gi|71895479	PMM2	28.59/5.79	9	1/2	56			9.06
122	triosephosphate isomerase	gi|45382061	TPI1	26.83/6.71	55	6/14	105			8.70
123	triosephosphate isomerase	gi|45382061	TPI1	26.83/6.71	75	13/23	276			8.66
130	alpha-enolase	gi|46048768	ENO1	47.62/6.17	39	9/22	943			-8.76
132	malate dehydrogenase, cytoplasmic	gi|57530355	MDH1	36.75/6.92	27	1/11	237			-8.76
133	ubiquinol-cytochrome c reductase	gi|50754375	UQCRH	53.41/6.58	26	2/16	430			-9.99
144	L-lactate dehydrogenase A chain	gi|45384208	LDHA	36.78/7.75	36	6/15	271			1.14
145	alpha-enolase	gi|46048769	ENO1	47.62/6.17	49	15/33	644			1.35
**Protein and amino acid metabolism**										
1	elongation factor 2	gi|45382453	EEF2	96.34/6.40	39	24/53	387	8.34		
2	glycine dehydrogenase [decarboxylating], mitochondrial precursor	gi|45383510	GLDC	111.78/7.55	19	9/22	246	7.93		
3	methylcrotonoyl-CoA carboxylase subunit alpha, mitochondrial	gi|57529595	MCCC1	78.88/6.51	17	3/14	95	6.33	6.75	7.20
8	cytosol aminopeptidase	gi|71897015	LAP3	56.92/8.38	27	7/14	239	10.50		
21	guanidinoacetate N-methyltransferase	gi|118103242	GAMT	17.46/6.59	48	13/23	551	9.36		
26	SH3 domain-binding glutamic acid-rich-like protein	gi|60302796	Q5F3C9	12.79/5.07	90	1/22	325	8.54	7.76	7.81
35	cathepsin B precursor	gi|46195455	CTSB	38.48/5.74	27	6/21	530	-9.02		
43	glutamine synthetase	gi|45382781	GLUL	42.75/6.38	11	1/8	108	1.39		
50	sarcosine dehydrogenase, mitochondrial	gi|363740469	SARDH	102.26/6.42	27	4/30	633	8.34	-7.10	
51	dimethylglycine dehydrogenase, mitochondrial	gi|363744224	DMGDH	96.68/7.33	40	3/41	1091	7.93	-6.91	
52	histidine ammonia-lyase	gi|45383354	HAL	73.54/6.17	31	3/26	590		-7.52	
53	elongation factor 2	gi|45382453	EEF2	96.34/6.40	25	2/27	499		-7.73	
54	glycine dehydrogenase [decarboxylating], mitochondrial precursor	gi|45383510	GLDC	113.26/7.55	23	7/21	122		-6.86	
55	elongation factor 2	gi|45382453	EEF2	96.34/6.40	21	6/19	90		-7.63	
56	ovoinhibitor precursor	gi|71895337	SPINK5	54.39/6.16	18	6/9	137		-7.53	
57	S-adenosylmethionine synthase isoform type-1	gi|313760551	MAT1A	44.24/6.28	30	7/14	137		-7.73	
58	fumarylacetoacetase (Fumarylacetoacetate hydrolase)	gi|50753071	FAA	46.80/7.31	43	18/49	259		-9.88	
64	guanidinoacetate N-methyltransferase	gi|118103242	GAMT	17.46/6.59	35	4/ 12	249		-8.71	
69	glutamine synthetase	gi|45382781	GLUL	42.75/6.38	29	6/15	171		-9.73	
74	alanyl-tRNA synthetase, cytoplasmic	gi|57524852	AARS	102.00/5.68	38	25/50	623		8.85	
78	trifunctional purine biosynthetic protein adenosine-3	gi|47825387	GART	107.56/7.51	26	6/26	132		7.07	
81	glutamine synthetase	gi|45382781	GLUL	42.75/6.38	40	8/24	171		9.64	
90	4-hydroxyphenylpyruvate dioxygenase	gi|363739843	HPD	45.05/6.41	59	5/27	374		-1.43	
93	eukaryotic translation initiation factor 3 subunit I	gi|256419027	EIF3I	36.87/5.38	32	2/13	338		1.02	
94	3-hydroxyanthranilate 3,4-dioxygenase	gi|118087959	HAAO	33.74/6.27	18	1/4	131		-1.82	
95	guanidinoacetate N-methyltransferase	gi|118103242	GAMT	17.46/6.59	44	7/18	370		1.17	
97	pterin-4-alpha-carbinolamine dehydratase	gi|45382483	PCBD1	12.05/6.04	69	4/20	415		-1.36	
103	S-adenosylmethionine synthase isoform type-1	gi|313760551	MAT1A	44.24/6.28	55	28/57	664		-2.67	
105	S-adenosylmethionine synthase isoform type-1	gi|313760551	MAT1A	44.24/6.28	10	3/5	94		1.11	
108	lysyl-tRNA synthetase	gi|71895483	KARS	68.33/5.89	36	3/28	809			6.775
110	phenylalanine-4-hydroxylase	gi|47604920	PAH	51.51/6.49	53	8/42	1247			8.80
139	cytosolic non-specific dipeptidase	gi|57530409	CNDP2	53.39/5.71	39	4/21	690			1.66
140	aldehyde dehydrogenase 9 family, member A1	gi|118094103	ALDH9A1	57.00/7.81	65	34/53	1215			1.13
**Nucleotide metabolism**										
19	adenosine 5-diphosphosugar pyrophosphatase	gi|118081976	GMPS	35.43/6.36	28	9/15	312	8.78		8.38
39	adenosine kinase	gi|57529848	ADK	40.46/6.06	20	3/8	96	-1.21		
40	dihydropyrimidinase	gi|118087274	DPYS	69.50/6.42	19	4/16	108	1.48		
61	adenosine 5-diphosphosugar pyrophosphatase	gi|118081976	GMPS	35.43/6.36	22	6/12	504		-9.03	
62	ribokinase	gi|118088003	RBKS	36.72/5.39	22	3/7	378		-8.01	
99	ribose-phosphate pyrophosphokinase 2	gi|57525515	PRPS2	36.04/6.37	58	31/53	865		-1.35	
125	nucleoside diphosphate kinase	gi|45384260	NME5	17.45/7.72	79	16/36	264			8.59
128	nucleolar protein B23/No38	gi|212456	NPM1	10.77/4.38	57	1/6	205			-8.94
135	p32 subunit of splicing factor SF2	gi|5509946	C1QBP	23.78/4.41	23	2/5	238			1.14
141	thiosulfate sulfurtransferase	gi|268370289	TST	33.09/6.80	52	22/34	503			-1.20
143	nucleoside diphosphate kinase	gi|2827446	NME5	17.54/7.11	66	32/45	1004			-1.33
**Fatty acid metabolism**										
27	fatty acid-binding protein	gi|45384320	FABP7	15.03/5.61	56	2/6	85	8.68	8.52	9.24
30	long-chain specific acyl-CoA dehydrogenase, mitochondrial	gi|57529797	ACADL	48.26/8.34	32	10/17	159	-10.58		
33	dodecenoyl-Coenzyme A delta isomerase	gi|118098151	ECI1	34.56/9.30	33	18/32	379	-8.52		-7.68
42	3-hydroxy-3-methylglutaryl-coenzyme A synthase	gi|118094097	HMGCS1	52.98/6.57	31	11/26	195	1.69		
48	fatty acid-binding protein	gi|45384320	FABP7	15.03/5.61	71	9/16	276	2.16		
65	sulfotransferase 1B	gi|118090275	SULT1B1	32.01/6.00	48	1/16	351		-6.71	
70	phosphatidylethanolamine-binding protein 1	gi|310772215	PEBP1	21.12/6.96	66	4/17	419		8.23	
82	3-ketoacyl-CoA thiolase, mitochondrial	gi|57529492	HADHA	42.17/8.02	18	3/7	89		10.03	
111	3-hydroxy-3-methylglutaryl-coenzyme A synthase	gi|118094097	HMGCS1	52.98/6.57	30	16/25	383			10.33
112	3-hydroxy-3-methylglutaryl-coenzyme A synthase	gi|118094097	HMGCS1	52.98/6.57	32	15/27	462			9.48
114	SEC14-like 2	gi|50756739	SEC14L3	46.94/6.73	60	24/43	520			10.40
116	phosphatidylinositol transfer protein beta isoform	gi|86129444	PITPNB	30.86/5.63	71	12/27	224			7.01
**Antioxidants**										
6	epoxide hydrolase 2	gi|45384320	EPHX2	63.72/5.89	45	17/29	448	8.37		
46	peroxiredoxin-6	gi|57529797	PRDX6	25.08/5.72	58	4/35	793	1.00		
68	thioredoxin	gi|118098151	TXNRD1	11.98/5.10	49	1/12	285		-8.97	
76	serum albumin precursor	gi|118094097	ALB	71.87/5.51	26	1/14	362		8.70	
77	serum albumin precursor	gi|45384320	ALB	71.87/5.51	48	18/31	455		9.23	9.39
86	glutathione S-transferase 2	gi|118090275	GSTM2	26.05/6.85	80	9/33	746		8.86	
118	thioredoxin-like protein	gi|310772215	TXNL1	32.73/4.94	40	5/18	131			8.55
124	peroxiredoxin-1 isoform 1	gi|57529492	PRDX1	22.53/8.24	40	2/15	390			8.19
126	thioredoxin	gi|118094097	TXNRD1	11.98/5.10	49	2/6	61			8.82
127	thioredoxin	gi|118094097	TXNRD1	11.98/5.10	32	1/3	67			8.22
136	protein disulfide-isomerase A3 precursor	gi|50756739	PDIA3	56.55/5.76	32	2/16	507			2.25
142	retinal dehydrogenase 1	gi|86129444	ALDH1A1	56.40/7.49	51	24/35	615			1.30
**Cell structure**										
12	alpha-tropomyosin 2	gi|27465053	TPM1	32.85/4.65	40	8/17	164	7.73		
15	protein syndesmos	gi|45382147	NUDT16L1	33.98/5.74	48	12/33	277	7.24		
28	lamin-A	gi|45384214	LMNA	73.35/6.50	14	1/11	373	-8.00		
36	mitochondrial inner membrane protein	gi|57530041	IMMT	79.54/5.72	31	2/31	745	1.24		
37	mitochondrial inner membrane protein	gi|57530041	IMMT	79.54/5.72	24	2/28	479	1.02		
38	desmin	gi|2959450	DES	51.69/5.30	53	12/35	272	1.46		
63	F-actin-capping protein subunit alpha-1	gi|45382905	CAPZA1	33.11/5.43	53	3/15	499		-8.42	
66	translationally-controlled tumor protein homolog	gi|45382329	TPT1	19.69/4.90	90	12/23	372		-7.95	
67	glia maturation factor beta	gi|71894963	GMFB	16.88/5.19	28	1/4	144		-8.10	
71	translationally-controlled tumor protein homolog	gi|45382329	TPT1	19.69/4.90	50	8/24	190		8.19	
75	mitochondrial inner membrane protein	gi|57530041	IMMT	79.54/5.72	40	15/36	1360		6.91	
79	lamin-A	gi|45384214	LMNA	73.35/6.50	58	24/44	512		7.76	
92	alpha-tropomyosin	gi|211109	TPM1	32.81/4.75	15	2/4	177		1.61	
109	coronin-1C	gi|86129440	CORO1C	53.74/6.22	39	20/34	244			6.78
**Protein folding**										
49	10 kDa heat shock protein, mitochondrial	gi|45384204	HSPE1	11.13/8.68	82	1/20	438	-1.5892		
59	cyclophilin	gi|118089782	PPIA	39.80/5.61	27	4/16	95		-7.12	
106	T-complex protein 1 subunit alpha	gi|57530301	TCP1	61.06/5.66	34	9/18	181			8.83
107	T-complex protein 1 subunit alpha	gi|57530301	TCP1	61.06/5.66	43	11/32	1071			9.28
131	hsc70-interacting protein	gi|71896903	ST13	40.36/5.07	36	8/22	646			-6.97
134	peptidylprolyl isomerase A	gi|261490820	PPIA	18.08/8.29	67	9/28	194			-9.24
**Transporter**										
24	heme-binding protein 1	gi|71896913	HEBP1	21.26/5.76	34	3/7	88	7.85		
31	chloride intracellular channel protein 2	gi|71895359	CLIC2	28.46/5.39	42	4/19	492	-8.06		
119	coatomer subunit epsilon	gi|57530593	COPE	34.52/4.99	35	4/31	371			9.29
129	clathrin light chain A	gi|86129544	CLTA	23.86/4.42	40	6/12	189			-8.33
137	transitional endoplasmic reticulum ATPase	gi|113206112	VCP	89.95/5.14	54	21/60	496			1.04
**Unknown function**										
9	MGC82288 protein	gi|50729534		50.12/6.35	44	14/23	264	10.66		
13	protein PRRC1	gi|71894751	PRRC1	45.84/5.52	10	2/4	80	7.89		
20	hypothetical protein RCJMB04_5n23	gi|53129586	TPM3	28.80/4.69	47	12/24	305	9.33		
25	hypothetical protein	gi|118083300		10.04/4.17	14	2/4	58	9.29		
32	ES1 protein homolog, mitochondrial	gi|71895261	C1H21orf33	27.87/8.54	46	17/45	213	-9.54		
34	hypothetical protein	gi|118098539		28.31/7.63	42	3/28	647	-8.71		
41	hypothetical protein RCJMB04_1j22	gi|53127216		60.28/8.09	51	20/40	412	1.23		
60	hypothetical protein	gi|118087385		44.65/5.82	16	4/6	185		-8.23	
72	sorcin	gi|124249424	SRI	22.21/5.37	55	9/17	216		8.01	
73	sorcin	gi|124249425	SRI	22.21/5.37	24	2/5	51		8.76	
91	hypothetical protein RCJMB04_1g3	gi|53126716		33.27/5.60	46	5/19	579		-1.06	
96	hypothetical protein	gi|50800573		11.84/4.42	66	8/11	348		1.06	
100	LOC495096 protein isoform 3	gi|50734923		28.44/6.45	34	2/13	297		1.26	
101	thyroid hormone responsive spot 14 beta 2	gi|45826439	THRSPB	14.52/5.10	20	1/3	98		1.13	
113	hypothetical protein	gi|50728520		48.21/6.29	42	15/30	430			10.66
115	LOC495029 protein	gi|118098511		45.06/6.55	63	26/45	716			9.86
117	MGC83663 protein	gi|118093845		51.57/6.30	30	6/18	730			8.70
138	MGC82230 protein	gi|50756617		43.26/5.76	61	11/33	1036			1.03

^a^ Spot no. corresponds to the number of protein spots in Fig 1. Protein name is given when proteins were identified by LC-Chip ESI-QTOF MS. Accession no. is the unique number given to mark the entry of a protein in the database NCBInr. Theoretical molecular weight (*Mr*) and isoelectric point (*p*I) of the identified proteins are retrieved from the protein database of NCBInr (S1 Table). Sequence coverage is the ratio of the number of amino acids in every peptide that matches with the mass spectrum divided by the total number of amino acids in the protein sequence. Matched peptide is the number of paring an experimental fragmentation spectrum to a theoretical segment of protein and searched peptide is the total searched peptide. Mascot scores are derived from ion scores as a non-probabilistic basis for ranking protein hits.

### Qualitative comparisons of proteins with differential abundance

**Fig 2:**
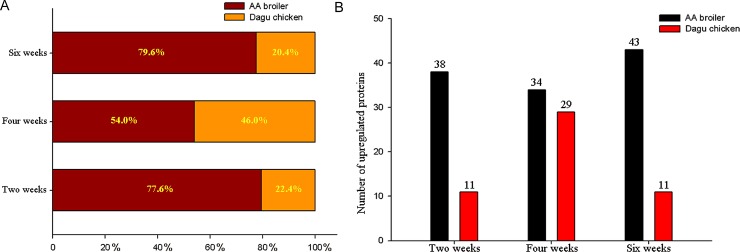
Comparisons proteins with higher abundance in the livers between the AA broiler and Big Bone chickens at 2, 4 and 6 weeks, respectively. A represents the percentage of proteins with increased abundance, B represents the numbers of proteins with increased abundance.

In general, the liver protein profiles between AA broiler and Big Bone chickens during three age stages were similar. However, some protein spots displayed obvious differences in abundance. As mentioned previously, a total of 49, 63, and 54 proteins were differentially expressed in AA broiler and Big Bone chickens at 2, 4, and 6 weeks, respectively. From the 49 identified proteins at 2 weeks, 38 (77.6%) and 11 (22.4%) had increased abundance in the AA broiler and the Big Bone chickens, respectively. Similarly at 4 weeks, 34 (54.0%) and 29 (46.0%) proteins had higher abundance in the AA broiler and the Big Bone chickens, respectively. Also at 6 weeks, the AA broiler and the Big Bone chickens differentially expressed 43 (79.6%) and 11(20.4%) proteins, respectively (Fig 2A and 2B). On average, the abundance for 115 (69.3%) proteins was higher in the AA broiler and that for 51 (30.7%) was higher in the Big Bone chickens.

### Classification of proteins with differential abundance

**Fig 3:**
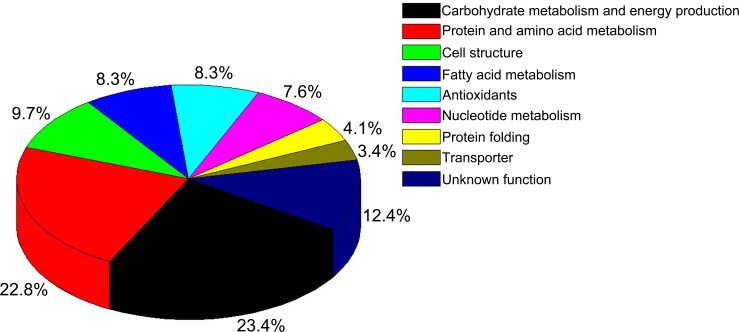
Functional classification of the proteins with differential abundance identified in the livers of AA broiler and Big Bone chickens at 2, 4 and 6 weeks.

Based on biological processes in the database annotations, the identified differential proteins between the AA broiler and the Big Bone chickens were classified into nine main functional categories, including carbohydrate metabolism and energy production (23.4%), protein and amino acid metabolism (22.8%), cell structure (9.7%), fatty acid metabolism (8.3%), antioxidants (8.3%), nucleotide metabolism (7.6%), protein folding (4.1%), transporters (3.4%), and unknown function (12.4%) (Fig 3).

### Qualitative comparisons of differentially expressed proteins

**Fig 4:**
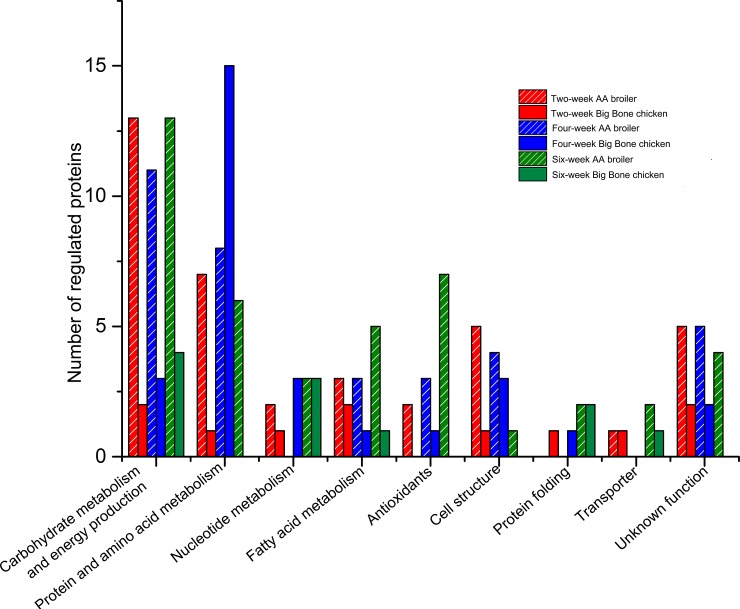
Comparisons of functional classification of proteins with higher abundance in the livers between the AA broiler and Big Bone chickens at 2, 4 and 6 weeks.

Interestingly, the higher abundance proteins at the different stages in the two breeds showed distinct functional categories (Fig 4). The number of proteins with higher abundance in the AA broilers liver was greater than in the Big Bone chickens liver at three age stages, which were mainly involved in carbohydrate metabolism and energy production, fatty acid metabolism, antioxidants and cell structure.

**Fig 5:**
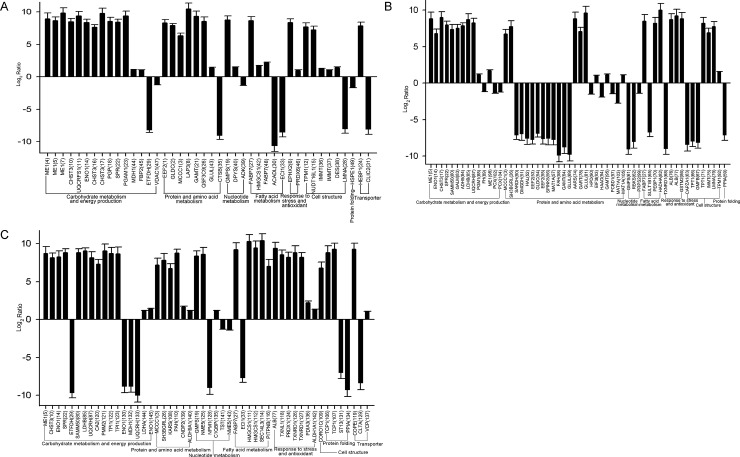
Quantitative comparisons of differentially expressed proteins in the livers of AA broiler and Big Bone chickens at 2, 4 and 6 weeks. The ratios of the protein abundance (AA broilers to Big Bone chickens) are transformed, and the protein spots with |log_2_ ratio|≥1 (p≤0.05) are selected as the differentially expressed proteins. A, B and C represent differentially expressed proteins at 2, 4 and 6 weeks, respectively.

**Fig 6:**
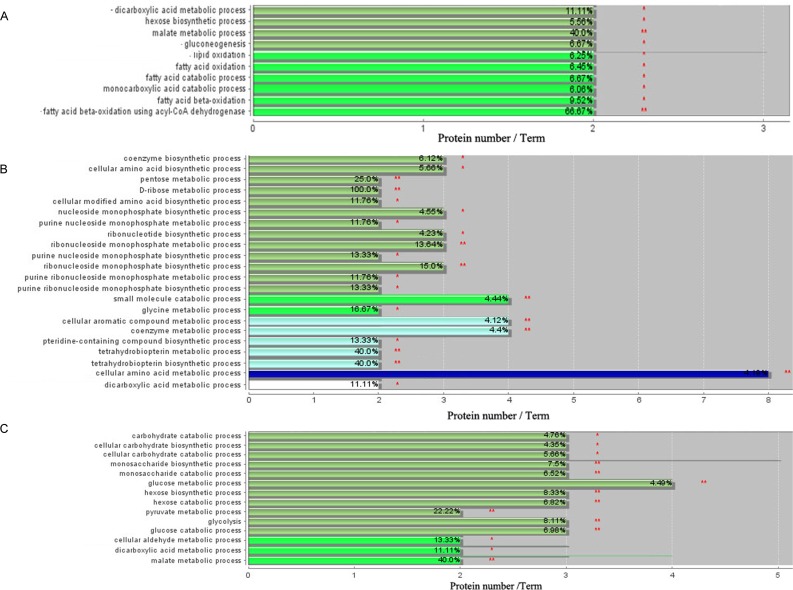
Functional enrichment analysis of the proteins of differential abundance in the livers between AA broiler and Big Bone chickens at 2, 4 and 6 weeks using ClueGO software. * and ** mean p < 0.05 and p < 0.01 levels of significance. A, B and C represent enrichment analysis of differentially expressed proteins at 2, 4 and 6 weeks, respectively.

The relative abundance between the two breeds was shown by Fig 5A, 5B and 5C. Enrichment analysis of the proteomes at 2 weeks showed that two major functional groups, namely carbohydrate metabolism and fatty acid metabolism, were significantly enriched (Fig 6A). Similarly, two main functional groups, including protein and amino acid metabolism and nucleotide metabolism, were enriched at 4 weeks (Fig 6B). At 6 weeks, the functional group carbohydrate metabolism was significantly enriched (Fig 6C).

### Biological network analysis

**Fig 7:**
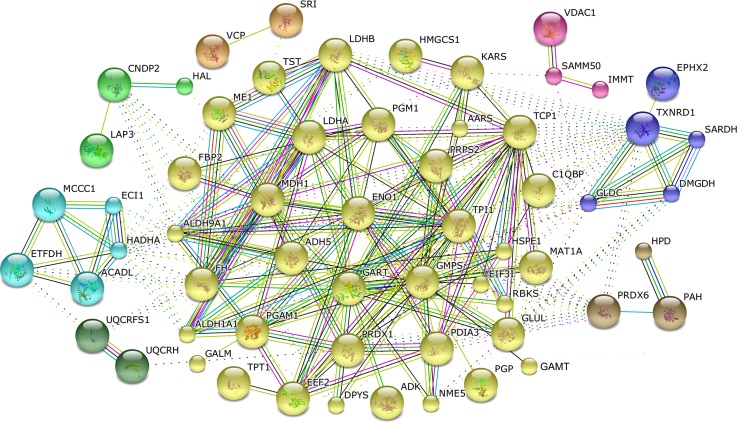
Biological interaction network of the proteins of differential abundance in the livers of AA broiler and Big Bone chickens at 2, 4 and 6 weeks. Red lines indicate fusion evidence, green lines indicate neighborhood evidence, blue lines indicate co-occurrence evidence, purple lines indicate experimental evidence, yellow lines indicate text mining evidence, light blue lines indicate database evidence and black lines indicate co-expression evidence.

Proteins are fundamental parts of living cells and cellular functions are mainly carried out by protein complexes. Using the online tool STRING 10.0, sixty proteins were recognized as nodes in biological interaction networks, in which eight clusters were enriched and connected by cytosolic non-specific dipeptidase (CNDP2), 3-ketoacyl-CoA thiolase (HADHA), electron transfer flavoprotein-ubiquinone oxidoreductase (ETFDH), ubiquinol—cytochrome c reductase (UQCRFS1), ubiquinol-cytochrome c reductase hinge protein (UQCRH), peroxiredoxin-6 (PRDX6), glycine dehydrogenase (GLDC), thioredoxin (TXNRD1), sorting and assembly machinery component 50 (SAMM50), fructose 1,6-bisphosphatase (FBP2) and aldehyde dehydrogenase 9 family (ALDH9A1) (Fig 7). The biggest cluster mainly involved in glycolysis/gluconeogenesis, carbon metabolism and amino acid metabolism pathway by the interaction between 37 proteins.

### Validation of proteins of differential abundance by qPCR

**Fig 8:**
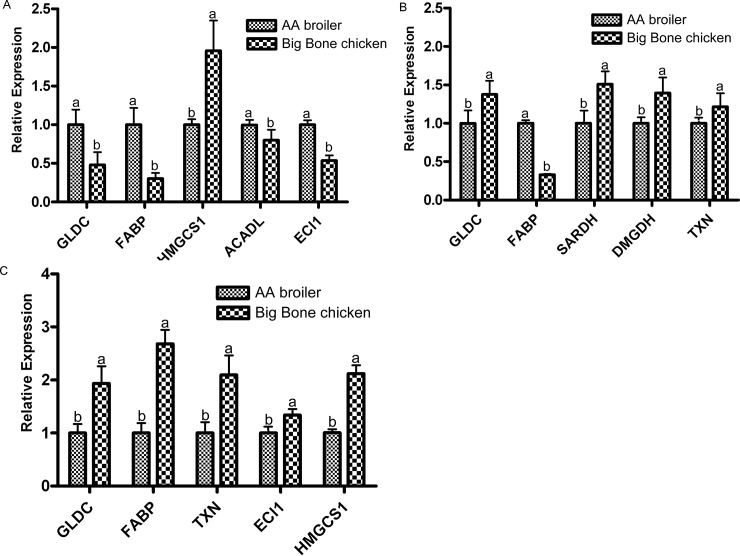
Validation using qPCR of proteins of differential abundance at the mRNA level in the livers between the AA broiler and Big Bone chickens at 2, 4 and 6 weeks. Samples were normalized with the reference genes β-*actin*. A, B and C represent differentially expressed proteins at the mRNA level at 2, 4 and 6 weeks, respectively.

Of the liver proteins with differential abundance at 2 weeks, 4 weeks and 6 weeks, proteins that played an important role in nutrient metabolism (amino acid and lipid metabolism) and antioxidants were selected to validate their expression at the level of mRNA (Fig 8). At 2 weeks (Fig 8A), GLDC (spot 2) and FABP (spots 27 and 48) at the protein levels were consistent with their mRNA expression levels; but HMSC1 (spot 42), ACADL (spot 30) and ECI1 (spot 33) showed an inconsistent pattern between the mRNA expression and protein abundance level. At 4 weeks (Fig 8B), GLDC (spot 54), FABP (spot 27), SARDH (spot 50), DMGDH (spot 51) and TXNRD1 (spot 168) were consistent with their mRNA expression levels. The similar expression pattern at the transcript level indicates a prospective opportunity for reverse genetic research through gene manipulation. At 6 weeks (Fig 8C), ECI1 (spot 33) at the protein level were consistent with their mRNA expression levels; however, FABP (spot 27), HMSC1 (spots 111 and 112) and TXNRD1 (spots 126 and 127) showed an inconsistent pattern between the mRNA expression and protein abundance level.

## Discussion

This study compared differences in the proteome and the weight of the liver between AA broiler and Big Bone chickens at three different age stages. Average abdominal fat percentage and the liver weight showed significant differences between two breeds at four weeks of age. Moreover, AA broilers also showed the most differential proteome at four weeks compared to Big Bone chickens. Additionally, the number of proteins with increased abundance related to carbohydrate metabolism and energy production were higher in AA broilers than Big Bone chickens at three stages of age, which indicates breed differentiation of the two strains may be associated with differential abdominal fat deposition.

Malic enzyme (ME1, spot 4, 5, 7 and 98), ubiquinol-cytochrome C reductase (UQCRFS1, spot 11), enolase 1 (ENO1, spot 14), phosphoglycerate mutase 1 (PGAM1, spot 23), malate dehydrogenase (MDH1, spot 44), fructose-1, 6-bisphosphatase (FBP2, spot 45), phosphoglucomutase 1 (PGM1, spot 88), and triosephosphate isomerase (TPI1, spot 122 and 123) are key proteins which showed higher abundance in AA broiler chickens to mediate energy production. MDH1, FBP2, PGM1 and phosphoglycerate mutase 1 (PGAM1, spot 23) participate in the TCA cycle and UQCRFS1 (spot 11) is an electron transporter chain protein of the mitochondria to promote energy production. Although the TCA cycle is mainly involved in energy metabolism, it also produces intermediates to be converted to glucose, fatty acids, or non-essential amino acids [20]. Previously, malic enzyme gene expression in hepatic metabolism was found to be enhanced in fatter chicken varieties over lean chicken varieties [21], which was consistent with increased abundance for ME1 protein in AA broilers. Moreover, the higher abundance for pyruvate carboxylase (PCX, spot 102 and 104) and fumarate hydratase (FH, spot 89) in AA broilers at 4 weeks are the key enzymes involved in energy production and gluconeogenesis. Therefore, these differentially expressed proteins strongly suggest higher energy metabolism and enhanced lipid biosynthesis in AA broilers than that in Big Bone chicken.

The liver is a vital organ for dietary protein and amino acid metabolism. Protein synthesis is subject to regulation by eukaryotic initiation factor 2 and elongation factor 2 (EEF2, spot 1, 53 and 55) [22, 23]. The abundance of EEF2 was higher in the liver of AA broilers at two weeks of age and in Big Bone chickens at four weeks. The differential EEF2 abundance at different stages in the two breeds may affect the protein synthesis. At the same time, AA broilers had more proteins with increased abundance involved in amino acid metabolism than Big Bone chickens at two weeks, but less than Big Bone chicken at four weeks. Guanidinoacetate N-methyltransferase (GAMT, spot 21) was higher at -two weeks and GAMT (spot 64) was lower at four weeks in AA broilers, however the abundance of GAMT (spot 95) was significantly differential between Big Bone chickens and AA broilers at four weeks of age. These protein spots were differentially modified and played different roles in creatine biosynthesis between two breeds [24]. Furthermore at four weeks, Big Bone chickens had increased levels of glycine dehydrogenase (GLDC, spot 54), sarcosine dehydrogenase (SARDH, spot 50) and dimethylglycine dehydrogenase (DMGDH, spot 51), which are all involved in glycine metabolism [25, 26]. This enhanced glycine metabolism may contribute to the better meat quality in Big Bone chickens because glycine is one of most important flavor substances.

Previous studies demonstrated differential lipid metabolism between fat and lean broiler breeds [5, 9, 27]. Specifically, this study found that AA broilers had enhanced levels of fatty acid-binding protein (FABP7, spot 27 and 48), enzyme HMG-CoA synthase 1 (HMGCS1, spot 42, 111 and 112), phosphatidylinositol transfer protein (PITPNB, spot 116) and lipid binding protein SEC14-Like 2 (SEC14L3, spot 114). This fatty acid-binding protein is involved in intracellular fatty acid movement and its abundance has been associated with fat deposition in chickens [28–30]. HMGCS1 is a rate-limiting enzyme for ketone body formation from fatty acids in mitochondria [31]. PITPNB catalyzes the transfer of phosphatidylinositol and phosphatidylcholine between membranes for lipid delivery [32]. SEC14L3 stimulates squalene monooxygenase in the cholesterol biosynthetic pathway [33]. The increased abundance of these proteins suggest that AA broilers have enhanced lipid metabolism. On the contrary, Big Bone chickens had higher levels of acyl-CoA dehydrogenase long chain (ACADL, spot 30) and enoyl CoA isomerase 1 (ECI1, spot 33), both of which are involved in the beta-oxidation of fatty acids [34, 35]. Thus the enhanced lipid degradation in Big Bone chickens may be related to their reduced abdominal fat percentage.

Nucleotides are used in a wide variety of cellular metabolism and are fundamental for cellular functions [36]. The abundance for ribose-phosphate pyrophosphokinase 2 (PRPS2, spot 99) was increased in AA broilers liver at two weeks and four weeks of age. PRPS2 catalyzes the synthesis of phosphoribosyl pyrophosphate in purine nucleotide synthesis [37, 38]. In contrast, adenosine kinase (ADK, spot 39) was higher abundance in Big Bone chickens at two weeks of age and its function is known to be associated with liver disease [39]. Moreover, ribokinase (RBKS, spot 62) was higher abundance in Big Bone chickens at four weeks. RBKS belongs to the transferase family and participates in the pentose phosphate pathway. Nucleolar protein B23/No38 (NPM1, spot 128) and thiosulfate sulfurtransferase (TST, spot 141) were higher abundance in Big Bone chickens at six weeks. Thus, the differential nucleotide metabolism may contribute to variation in body weight between AA broiler and Big Bone chickens.

Additionally, differential abundance of liver proteins was observed, including those involved in the antioxidant system, protein folding, cytoskeleton, and transport proteins. These proteins may also play a role in regulating the size of the liver and the body weight of different breeds. Nevertheless, proteins function together in the context of networks through protein-protein interactions [40]. Protein associations involved in the development of the two breeds was analyzed by STRING. The majority of proteins identified were related to carbohydrate metabolism and energy production; approximately 31% of the biological interaction networks, followed by proteins in amino acid and protein metabolism (29%), antioxidants (13%) and nucleotide metabolism (13%). Moreover, proteins associated with protein folding, cell structure and fatty acid metabolism were nodes in the biological interaction networks, indicating that these proteins play a role in the construction and function of the liver of chickens. Some of the key node proteins that were highly linked in the BIN were validated at a gene level. GLDC, FABP, SARDH, DMGDH and TXNRD1 at different age stages were consistent or in consistent with mRNA levels may represent potential targets for genetic manipulation.

In conclusion, proteomic analysis of two unique chicken breeds was performed and differences in multiple metabolic pathways were identified between modern broilers and a local chicken breed. These findings can be used in the future to improve meat quality in commercial chicken supplies as the demand increases for not only greater amounts of food but better tasting meat.

## Supporting Information

S1 Fig The quality of RNA and qPCR reaction curve of the differentially expressed proteins at the mRNA level in the liver of AA broiler and Big Bone chickens. (DOCX)

S1 Table Peptides identified from the liver of AA broiler and Big Bone chickens by mass spectrometry. (XLSX)

S2 Table The primer sequences used for qPCR analysis of the differentially expressed proteins in the liver of AA broiler and Big Bone chickens. (DOCX)
