# Supplementary material for: Molecular Differences in Hepatic Metabolism between AA Broiler and Big Bone Chickens: A Proteomic Study
Source: PLoS One. 2016 Oct 19;11(10):e0164702. doi: 10.1371/journal.pone.0164702 (PMC5070854; doi:10.1371/journal.pone.0164702)

1. **Agarose gel electrophoresis image of total RNA**

**
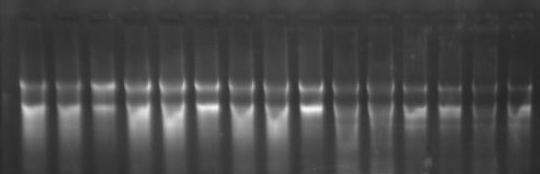

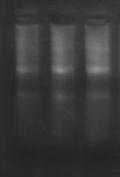
**

1. **Total RNA solution**

| **Sample** | **OD_260/280_** | **Con.(ng/ul)** |
| --- | --- | --- |
| 2WAA1 | 1.95 | 1984 |
| 2WAA2 | 2.00 | 2010 |
| 2WAA3 | 1.98 | 1824 |
| 2WD1 | 1.84 | 2186 |
| 2WD2 | 1.99 | 2800 |
| 2WD3 | 1.84 | 2200 |
| 4WAA1 | 1.85 | 1922 |
| 4WAA2 | 1.95 | 1751 |
| 4WAA3 | 1.96 | 1640 |
| 4WD1 | 1.92 | 2050 |
| 4WD2 | 1.99 | 1365 |
| 4WD3 | 1.95 | 1400 |
| 6WAA1 | 1.93 | 2000 |
| 6WAA2 | 1.95 | 1920 |
| 6WAA3 | 1.90 | 1890 |
| 6WD1 | 1.90 | 1570 |
| 6WD2 | 1.90 | 1746 |
| 6WD3 | 1.93 | 1757 |

1. **Reaction Curve**

**3. 1 FABP gene amplification and melting curve**


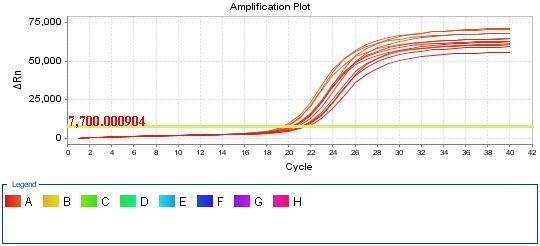

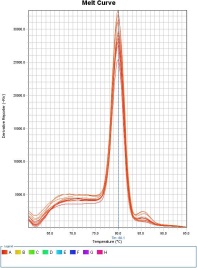


**3.2 HMGCS1 gene amplification and melting curve**


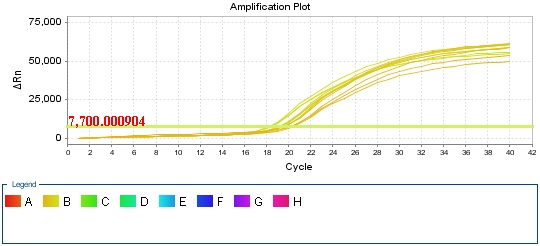

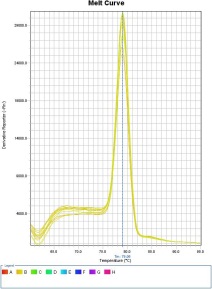


**3.3 ACADL gene amplification and melting curve**


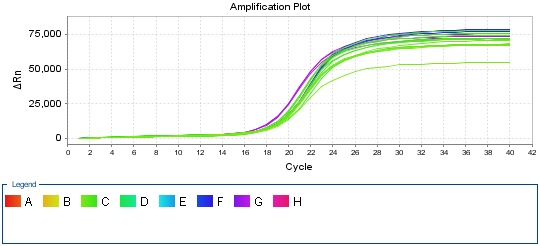

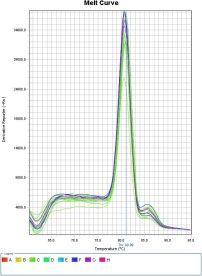


**3.4 ECl1 gene amplification and melting curve**


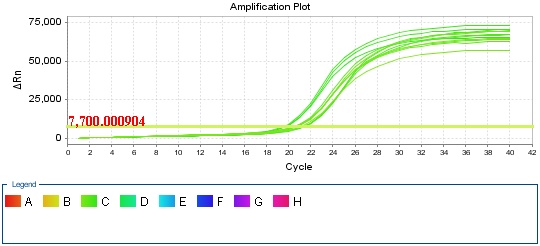

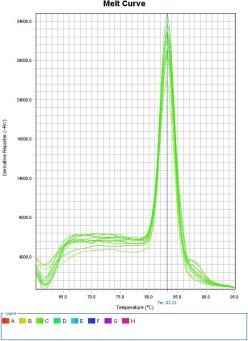


**3.5 SARDH gene amplification and melting curve**


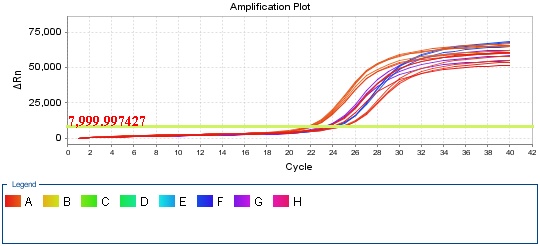

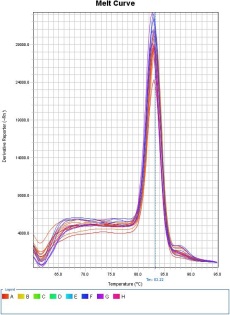


**3. 6 DMGDH gene amplification and melting curve**


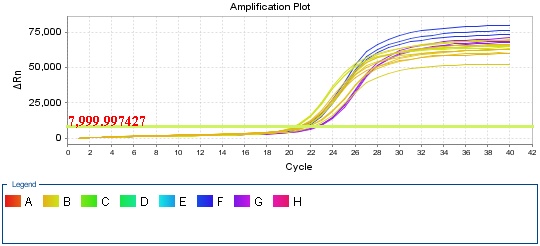

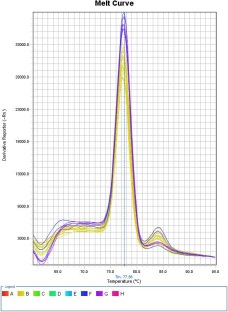


**3. 7 TXNRD1 gene amplification and melting curve**


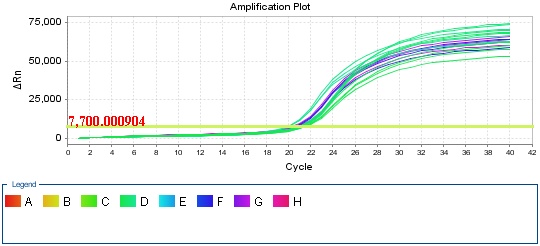

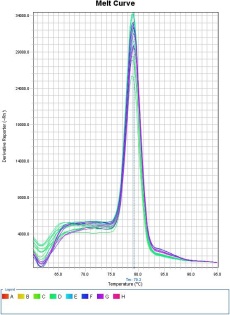


**3. 8 GLDC gene amplification and melting curve**


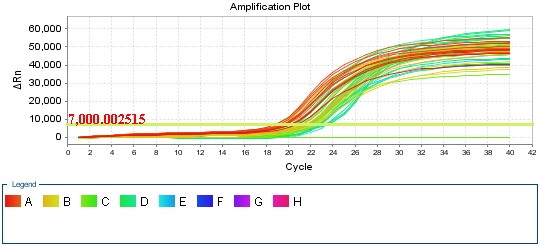

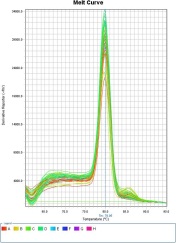


**3. 9 ACTB gene amplification and melting curve**


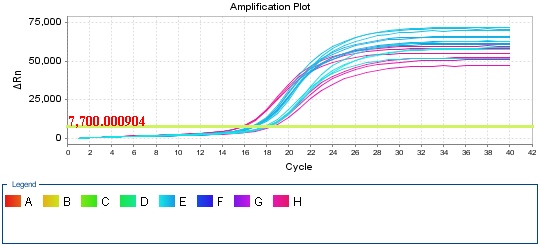

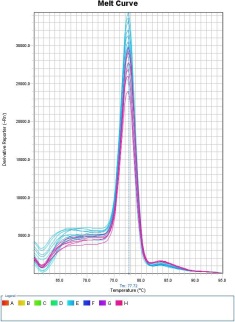

Supplement: S1 Fig — (DOCX) [file pone.0164702.s001.docx]
