# Supplementary material for: Molecular Differences in Hepatic Metabolism between AA Broiler and Big Bone Chickens: A Proteomic Study
Source: PLoS One. 2016 Oct 19;11(10):e0164702. doi: 10.1371/journal.pone.0164702 (PMC5070854; doi:10.1371/journal.pone.0164702)
Supplement: S2 Table — (DOCX) [file pone.0164702.s003.docx]

**Table S1. The primer sequences used for qPCR analysis of the differentially expressed proteins of the liver of AA broiler and Big Bone chickens**

| **Protein Spot no.** | **Symbol ID** | **Gene ID** | **Gene name** | **Primer sequence (5′→3′)** | **Product**  **size (bp)** | ***T*m**  **(°C)** |
| --- | --- | --- | --- | --- | --- | --- |
| 30 | ACADL | NM_001006511.2 | long-chain specific acyl-CoA dehydrogenase | GGTTTGCTGGGTGTTGCTAT | 112 | 60 |
|  |  |  |  | ATCCTGGGCCAGTACAGTTG |  |  |
| 51 | DMGDH | XM_003642954.1 | dimethylglycine dehydrogenase | CTGTCACCCTTTGGCAAGTT | 99 | 60 |
|  |  |  |  | ATTTGTGGAGCCAACCTTTG |  |  |
| 42, 111, 112 | HMGCS1 | NM_205411.1 | 3-hydroxy-3-methylglutaryl-coenzyme A synthase | TCTGCTCTGACCGAGAGGAT | 117 | 60 |
|  |  |  |  | TTGTCTCCGTTCCAACTTCC |  |  |
| 50 | SARDH | XM_415428.3 | sarcosine dehydrogenase | TCAAATCCAGCATTCCCTTC | 108 | 60 |
|  |  |  |  | ACATTGGCACCTTCTCATCC |  |  |
| 68,126,127 | TXNRD1 | NM_205453.1 | thioredoxin | ATGTGGATGATGCCCAAGAT | 115 | 60 |
|  |  |  |  | TTCTCTTTATTGGCCCCAGA |  |  |
| 33 | ECl1 | NM_001277585.1 | dodecenoyl-Coenzyme A delta isomerase | CTCAGCCTGGACTTCCTCAC | 108 | 60 |
|  |  |  |  | TGAGAAGACTCTGGGGATGG |  |  |
| 27,48 | FABP7 | NM_205308.2 | fatty acid-binding protein | CGTGACTAAGCCCACAGTGA | 102 | 60 |
|  |  |  |  | TCGCCGAGTTTAAAGCTGAT |  |  |
| 2,54 | GLDC | NM_204322.1 | glycine dehydrogenase | GCAGCATTCTTTGCTGTCAA | 114 | 60 |
|  |  |  |  | TCGGGTTTGTAAAGCCAGTC |  |  |
| Ref | ACTB | [NM_205518.1](http://www.ncbi.nlm.nih.gov/nuccore/NM_205518.1) | β-actin | AACACCCACACCCCTGTGAT | 100 | 60 |
|  |  |  |  | TGAGTCAAGCGCCAAAAGAA |  |  |
